# Production of Conjugated Linoleic and Conjugated *α*-Linolenic Acid in a Reconstituted Skim Milk-Based Medium by Bifidobacterial Strains Isolated from Human Breast Milk

**DOI:** 10.1155/2014/725406

**Published:** 2014-07-06

**Authors:** María Antonia Villar-Tajadura, Luis Miguel Rodríguez-Alcalá, Virginia Martín, Aránzazu Gómez de Segura, Juan Miguel Rodríguez, Teresa Requena, Javier Fontecha

**Affiliations:** ^1^Departamento de Bioactividad y Análisis de Alimentos, Instituto de Investigación en Ciencias de la Alimentación CIAL (CSIC-UAM) CEI UAM+CSIC, C/Nicolás Cabrera 9, Campus de la Universidad Autónoma de Madrid (UAM), 28049 Madrid, Spain; ^2^Departamento de Nutrición, Bromatología y Tecnología de los Alimentos, Universidad Complutense de Madrid, 28040 Madrid, Spain; ^3^Departamento de Biotecnología y Microbiología de Alimentos, Instituto de Investigación en Ciencias de la Alimentación CIAL (CSIC-UAM) CEI UAM+CSIC, C/Nicolás Cabrera 9, Campus de la Universidad Autónoma de Madrid (UAM), 28049 Madrid, Spain

## Abstract

Eight bifidobacterial strains isolated from human breast milk have been tested for their abilities to convert linoleic acid (LA) and *α*-linolenic acid (LNA) to conjugated linoleic acid (CLA) and conjugated *α*-linolenic acid (CLNA), respectively. These bioactive lipids display important properties that may contribute to the maintenance and improvement human health. Three selected *Bifidobacterium breve* strains produced CLA from LA and CLNA from LNA in MRS (160–170 and 210–230 *μ*g mL^−1^, resp.) and, also, in reconstituted skim milk (75–95 and 210–244 *μ*g mL^−1^, resp.). These bifidobacterial strains were also able to simultaneously produce both CLA (90–105 *μ*g mL^−1^) and CLNA (290–320 *μ*g mL^−1^) in reconstituted skim milk. Globally, our findings suggest that these bifidobacterial strains are potential candidates for the design of new fermented dairy products naturally containing very high concentrations of these bioactive lipids. To our knowledge, this is the first study describing CLNA production and coproduction of CLA and CLNA by *Bifidobacterium breve* strains isolated from human milk in reconstituted skim milk.

## 1. Introduction

Conjugated linoleic acid (CLA) and conjugated *α*-linolenic acid (CLNA) are bioactive lipids with potentially relevant benefits to human health. They have been shown to have* in vitro* and* in vivo* anticarcinogenic, antiatherogenic, anti-inflammatory, and antidiabetic activities and ability to reduce body fat [1, 2].

In relation to CLA, the bioactive isomer* cis 9, trans 11* CLA is the most abundant in the diet, constituting more than 90% of the total CLA content in milk fat [2, 3]. On the other hand, the CLNA isomers resulting from the metabolism of intestinal and rumen bacteria are* cis 9, trans 11, cis 15* CLNA and* trans 9, trans 11, cis 15* CLNA [1]. The predominant isomer is* cis 9, trans 11, cis 15* CLNA, which has been detected at low concentrations in milk fat [4, 5]. Presence of CLA and CLNA isomers in ruminant milk fat are the result of microbial partial biohydrogenation of dietary linoleic (LA) or *α*-linolenic (LNA) acid to stearic acid metabolism pathway in the rumen by the action of the linoleic acid isomerase [6]. CLA may also be formed through endogenous conversion of trans-vaccenic acid by the enzyme Δ^9^-desaturase in the mammary gland [7–9].

Nevertheless, as the current nutritional recommendations for whole fat dairy products are that their consumption should be limited, the CLA and CLNA content of human diet is too low for obtaining health beneficial effects. Therefore, a promising strategy to increase human intake of these bioactive lipids would be to include CLA and/or CLNA-producer bacteria in fermented dairy products. In the last years, several studies have reported that some lactic acid bacteria and bifidobacterial strains are able to efficiently convert LA to CLA in milk, milk-based media, and dairy products [10–12]. Moreover, other study demonstrated that a CLA-producing* Bifidobacterium breve* strain can be applied for the development of functional dairy products when used as a started culture [13]. In contrast, at present, we have not found studies showing CLNA production by bacteria in milk and dairy products. In this context, the aim of the present work was to evaluate the ability of some bifidobacterial strains isolated from human breast milk to produce CLA and/or CLNA when growing not only in MRS broth but also in reconstituted skim milk. In this study, we demonstrated that some* Bifidobacterium breve* strains are able to (co)produce CLA and CLNA in both media.

## 2. Material and Methods

### 2.1. Analytical Reagents

All reagents used in the lab procedures were of HPLC grade: hexane and sulphuric acid were obtained from Labscan (Dublin, Ireland), linoleic acid (C18:2* cis 9 cis 12*) from Sigma-Aldrich (St. Louis, MO, USA), linolenic acid (C18:3* cis 9 cis 12 cis 15*) from Nu-Chek Prep, Inc. (Elysian, USA), and high CLA content oil (Tonalin) from Cognis (Illertissen, Germany). LA and LNA were prepared as a 30000 *μ*g mL^−1^ stock solution containing 2% (w/v) Tween 80 (Scharlau, Sentmenat, Barcelona, Spain) and filter-sterilized through a 0.45 *μ*m-pore size membrane (Sarstedt, Nümbrecht, Germany).

### 2.2. Bacterial Strains, Growth Media, and Conditions

Eight bifidobacterial strains previously isolated from human milk [14, 15] were used in the study (Table 1). The bacterial strains were grown overnight at 37°C in MRS broth supplemented with 0.05% (w/v) L-cysteine-HCL (Sigma) and 0.1% (w/v) Tween-80 (MRS-Cys broth) under anaerobic conditions in an anaerobic station (Bactron II, Shellab, Cornelius, Oregon, USA). Three percent (v/v) of these cultures were transferred to fresh MRS-Cys broth (10 mL) containing free LA (500 *μ*g mL^−1^) and/or free LNA (500 *μ*g mL^−1^) and incubated at 37°C for 24 h under anaerobic conditions. The samples were analyzed when the bifidobacterial strains reach the early stationary phase, obtaining concentrations of ~1 × 10^9^ cfu mL^−1^. Only the strains that showed CLA production in MRS-Cys broth after an initial qualitative screening were subsequently tested for CLA and/or CLNA production in 10% skim milk (Scharlau, Sentmenat, Barcelona, Spain) supplemented with 0.05% (w/v) L-cysteine and 0.8% (w/v) casamino acids (milk-based medium), as described above.

Since the production of CLA and CLNA by the three selected* Bifidobacterium* strains in MRS-Cys was similar,* B. breve* M7-70 was chosen as the model strain for the subsequent assays. First, the sensitivity of* B. breve* M7-70 to different concentrations of LA or LNA (0, 250, 500, 1000, 1500, and 2000 *μ*g/mL) was evaluated in MRS-Cys broth since LA and LNA have antimicrobial properties. Then, this strain was submitted to a comparative analysis of CLA* versus* CLNA production at different times (0, 1, 2, 3, 4, 6, 8, 24, and 48 h) in MRS-Cys broth.

### 2.3. Qualitative Screening of CLA Producers by UV Spectroscopy Method

Lipid isolation from culture media was carried out using a chloroform/methanol (2 : 1, v/v) solution according to Folch method modified by [16]. The lipid residues obtained were subjected to a N_2_ flow and remained dissolved in chloroform at −20°C until spectrophotometric analysis. For this analysis, lipid extracts (200 *μ*L) from each sample were placed on a quartz 96-well plate (Hellma GmbH & Co. KG, Müllhein, Germany) and total CLA was quantified at a wavelength of 233 in a spectrophotometer (Varioskan Flash, Thermo Fisher Scientific, Waltham, MA, USA) according to [10]. Measurements were obtained in triplicate.

### 2.4. Quantitative Analysis of CLA and CLNA Production

The concentrations of CLA and CLNA in the culture media were determined using a direct transesterification method [17]. Heptadecanoic acid (C17:0; Sigma) was added to the samples as an internal standard. The fatty acid methyl esters (FAMEs) were dissolved in n-hexane and determined by gas liquid chromatography (GLC) in a chromatograph (Clarus 500, Pelkin Elmer, Beaconsfield, UK) equipped with a VF-23 column (30 m × 0.25 nm × 0.25 *μ*m, Varian, Middelburg, Netherlands). For GLC analysis, the initial temperature was 80°C. Then, the temperature was increased to 170°C at 30°C min^−1^, held at 170°C for 3 min, increased to 230°C at 30°C min^−1^ and, and finally held at 230°C for 7 min. Helium was used as the carrier gas at a pressure of 15 psig and with a split ratio of 1 : 50. The injection volume was 0.5 *μ*L and the analysis time was 15 min. Peaks were identified by comparing the retention times of CLA methylated standards (Nucheck, USA) and by gas chromatography-mass spectrometry (GC/MS). CLA and CLNA concentrations were expressed as *μ*g mL^−1^ and their conversion rates from LA and LNA were calculated using the formula [CLA/(CLA + LA)] × 100 and [CLNA/(CLNA + LNA)] × 100, respectively.

## 3. Results

The eight bifidobacterial strains assayed in this work were screened spectrophotometrically at A_233_ for CLA production from the LA added to the growth media, following the rapid method described by [10]. With this approach, a total of 3 strains (*Bifidobacterium breve* ZL12-28,* B. breve* 29M2, and* B. breve* M7-70) were identified as able to transform LA into CLA (Table 1).

**Figure 1:**
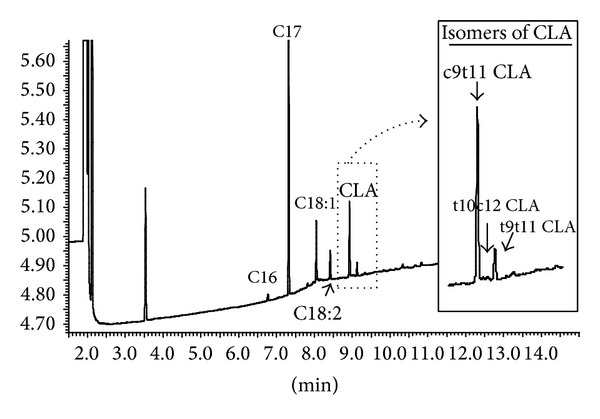
(a) Chromatogram profile assessed by gas chromatography of the fatty acid content present in the culture media obtained from* B. breve* M7-70 in MRS broth with 500 *μ*g mL^−1^ LA added as a substrate. (b) The insert shows a blow-up of the part of the chromatogram corresponding to the CLA isomers.

Subsequently, each of the three selected CLA-producing strains was assayed for CLA and CLNA production by GLC determination after incubation for 24 h at 37°C in MRS-Cys and skim milk (Tables 2 and 4). The concentration of CLA produced by the selected* Bifidobacterium* strains reached values above 158.7 *μ*g mL^−1^, indicating that the minimal conversion rate from the added LA was approximately 74%. The bacterial strains were able to produce different CLA isomers, such as* cis 9, trans 11* CLA,* trans 10, cis 12* CLA and* trans 9, trans 11* CLA (Figure 1); among them,* cis 9, trans 11* CLA (rumenic acid) was the most abundant isomer, accounting for more than 80% of the total CLA in all cases (Table 2). As it has been reported, many bacteria are inhibited by free long-chain fatty acids in the media [18]; however,* B. breve* M7-70 was able to grow in the presence of LA and LNA at concentrations up to 1500 and 500 *μ*g mL^−1^, respectively (Table 3).

**Figure 2:**
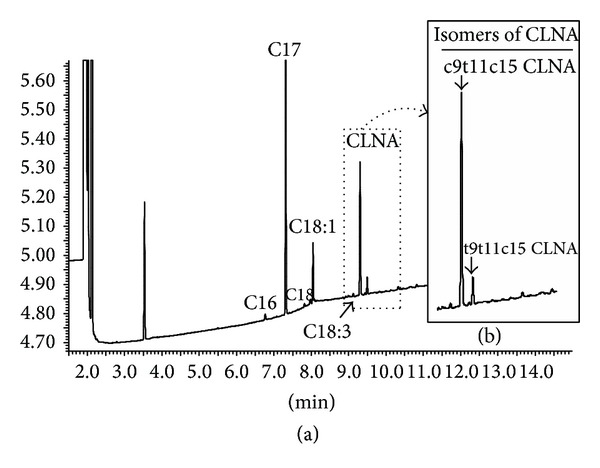
(a) Chromatogram profile assessed by gas chromatography of the fatty acid content present in the culture media obtained from* B. breve* M7-70 in MRS broth with 500 *μ*g mL^−1^ LNA as a substrate. (b) The insert shows a blow-up of the part of the chromatogram corresponding to the CLNA isomers.

**Figure 3:**
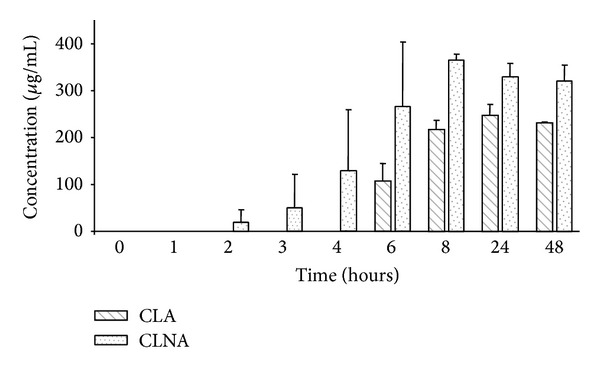
Comparative analysis of CLA versus CLNA production at different times. The* B. breve* M7-70 strain was incubated in MRS broth containing 500 *μ*g mL^−1^ free linoleic acid (LA) or 500 *μ*g mL^−1^ free linolenic acid (LNA) for 48 hours under anaerobic conditions. Samples were taken at the indicated times. Values are means ± SD of three independent experiments.

Production of CLNA in MRS-Cys by* B. breve* strains was higher than that of CLA since the concentrations found in the respective culture media were higher than 200 *μ*g mL^−1^, and the LNA to CLNA conversion rate was close to 100% (Table 2). Two CLNA isomers (*cis 9, trans 11, cis 15* CLNA and* trans 9, trans 11, cis 15* CLNA) could be detected in the chromatogram profiles (Figure 2), and* cis 9, trans 11, cis 15* CLNA (rumenic acid) accounted for approximately 80% of the total amount of CLNA in the cultures of the three strains (Table 2). The conversion of LNA to CLNA by* B. breve* M7-70 in MRS-Cys was faster than that of LA to CLA since CLNA production began after 2 h incubation at 37°C while CLA formation required at least 6 h of incubation to be detected (Figure 3).

Subsequently, the three selected bifidobacterial strains were assayed for CLA and/or CLNA production in the milk-based medium after 24 h incubation at 37°C. CLA production was above 75 *μ*g mL^−1^ for the three* B. breve* strains (Table 4). Both CLA production and LA conversion rate were lower in milk than in MRS-Cys. As expected, the predominant CLA isomer produced in reconstituted skim milk was* cis 9, trans 11* CLA, accounting for more than 80% of total CLA (Table 4).

All the selected strains were able to produce CLNA when growing in the milk-based medium. In this case, the concentration of CLNA produced by these bifidobacteria (200 *μ*g mL^−1^) and the conversion rates (~100%) were similar to those observed in MRS-Cys broth (Table 4). Rumenic acid was also the dominant isomer, accounting for approximately 90% of the CLNA total amount.

**Figure 4:**
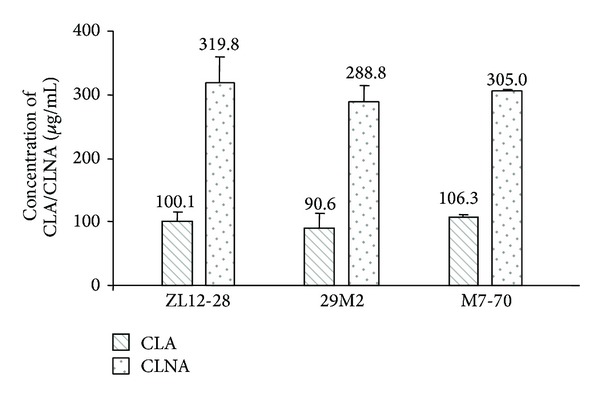
CLA/CLNA production by selected bacteria of screening. The cultures were incubated in reconstituted skim milk containing 500 *μ*g mL^−1^ free linoleic acid (LA) and 500 *μ*g mL^−1^ free linolenic acid (LNA) for 24 hours under anaerobic conditions. Values are means ± SD of three independent experiments.

Finally, the three selected bifidobacterial strains were tested for their ability to produce CLA and CLNA when both substrates (LA and LNA) were added to the milk-based medium. The three* B. breve* strains were able to simultaneously produce CLA and CLNA in these growth conditions (Figure 4). The CLA and CLNA concentrations produced were about 100 *μ*g mL^−1^ of CLA and 300 *μ*g mL^−1^ of CLNA (Figure 4). Interestingly, the production of both bioactive lipids was much higher when both substrates, LA and LNA, were present at the same time in the growing medium than when they were individually added (Table 4 and Figure 4).

## 4. Discussion

Bifidobacteria are numerically important members of the human gut microbiota and are believed to play a beneficial role in maintaining the health of the host. Some studies have suggested that infants with delayed bifidobacterial colonization and/or decreased bifidobacterial numbers may be more susceptible to a variety of gastrointestinal or allergic conditions [19, 20]. In these cases, the exogenous administration of selected bifidobacterial strains, alone or in combination with lactic acid bacteria, can reduce the incidence of such conditions [21–23]. Therefore, bifidobacteria are generally regarded as potentially probiotic microorganisms.

Recently, it has been shown that human milk is a source of live lactic acid bacteria and bifidobacteria to the infant gastrointestinal tract [24, 25]. If health benefits could be associated with bifidobacterial strains isolated from such biological fluid, then they would be immediately regarded as particularly attractive microorganisms since they would fulfil some of the main criteria generally recommended for human probiotics, such as human origin and adaptation to mucosal and dairy substrates [26, 27].

Since production of conjugated fatty acids by some bifidobacteria and lactic acid bacteria has been reported in the last years [1, 2, 12, 28], it is considered of interest to have human milk strains available with such capability. In this work, we describe the assayed conditions to select and characterize three* B. breve* strains with the ability to produce high amounts of CLA and CLNA during their growth in skim milk. It is not strange that the selection process ended with three strains belonging to the species* B. breve*, since this species seems to be particularly suited for production of these bioactive lipids [28–31]. The bioactive lipid production is a strain-specific attribute and, in fact, a non-CLA-producing* B. breve* strain (*B. breve* ZL12-22) was also found in this study.

It has been hypothesized that some bacteria can convert LA to CLA and LNA to CLNA as a detoxification mechanism [18, 28, 29, 32]. LA and LNA have antimicrobial activity and may alter the permeability of the plasmatic membrane of some Gram-positive bacteria [33]. It has been shown that both substrates have inhibitory effects on the growth of CLA and CLNA producing strains [28, 34]. Generally, free LNA is more toxic to bacteria than free LA, which is coincident with the results obtained in this work with* B. breve* M7-70. The hypothesis of the detoxification mechanism may explain why CLNA production by the three* B. breve* strains was more efficient than CLA production. It also could explain why the production of both CLA and CLNA was higher when both substrates were added together than when the strains were grown on each substrate separately (Table 4 and Figure 4).

At present, CLA is better characterised than CLNA. There are some research works that report the health-promoting properties of CLNA [1]. A recent study demonstrated the ability of a* B. breve* strain to produce CLNA and other conjugated fatty acids, such as conjugated *γ*-linolenic acid (CGLA) or conjugated stearidonic acid (CSA), from *γ*-linolenic acid and stearidonic acid, respectively [35]. Another work reported that* B. breve* NCIMB 702258 displayed a high conversion rate (79%) of *α*-linolenic acid into the* cis 9 trans 11 cis 15* CLNA isomer. This isomer can inhibit the growth of SW480 colon cancer cells [31]. All these mentioned works have described the CLNA production during growth of the strains in MRS broth. In contrast, the results of our study indicate that the selected* B. breve* strains were able to produce significant amounts of CLNA (200 *μ*g mL^−1^) in skim milk, with a conversion rate of LNA close to 100%. In addition, this study has demonstrated that* B. breve* strains were able to coproduce high levels of CLA and CLNA during growth in skim milk. Coproduction of CLA and CLNA in the described concentrations would be of relevance to increase the amount of these bioactive lipids in fermented milks. Globally, these results suggest that the three selected* B. breve* strains have a strong potential to be used as probiotics in fermented milks in order to increase human intake of CLA and CLNA. Further studies to optimize the culture conditions for increasing CLA and CLNA production at large-scale by fermentation processes would be needed. Additional studies will be also required to further elucidate the relevance of consumption of these bioactive lipids in human health.

## Figures and Tables

**Table 1:** CLA production level by the bifidobacterial strains screened in this study.

Strains	Production of CLA
*Bifidobacterium breve* ZL12-22	−∗
*Bifidobacterium breve* ZL12-28	+++
*Bifidobacterium breve* 29M2	+++
*Bifidobacterium breve* M7-70	+++
*Bifidobacterium infantis* ZL50-25	−
*Bifidobacterium longum* ZL89-79	−
*Bifidobacterium longum* ZL114-24A	−
*Bifidobacterium longum* ZL114-24B	−

*(−) No production; (+) slight production; (++) moderate production; (+++) high production.

**Table 2:** CLA and CLNA production (*μ*g mL^−1^) and conversion (%) of LA and LNA by the bifidobacterial strains when growing in MRS-Cys. Conversion calculated as ∑CLA/(LA + ∑CLA) × 100 and ∑CLNA/(LNA + ∑CLNA) × 100, respectively, based on the results of GC.

Strains	CLA	*cis 9 trans 11* (% CLA)	LA conversion (%)	CLNA	*cis 9 trans 11 cis 15* (% CLNA)	LNA conversion (%)
*B*. *breve* ZL12-28	170.6 ± 38.5 (a)	81.2 ± 0.8 (a)	74.6 ± 6.2 (a)	218.8 ± 39.0 (a)	82.7 ± 8.7 (a)	98.8 ± 0.6 (a)
*B*. *breve* 29M2	158.7 ± 48.3 (a)	85.6 ± 0.7 (b)	74.1 ± 7.0 (a)	211.6 ± 59.3 (a)	91.4 ± 3.2 (a)	95.7 ± 4.8 (a)
*B*. *breve* M7-70	170.3 ± 46.4 (a)	81.7 ± 1.0 (a)	77.8 ± 2.8 (a)	234.2 ± 81.5 (a)	85.7 ± 3.3 (a)	98.7 ± 0.9 (a)

Values are means of triplicate experiments and standard deviation (±SD).

Means in the same column with different lowercase letters are significant at *P* < 0.05.

**Table 3:** Growth of *B. breve* M7-70 in the presence of different concentrations of LA and LNA.

Concentration (mg mL^−1^)	LA	LNA
0	+++∗	+++
0.25	++	+
0.50	++	+
1.0	+	−
1.5	+	−
2.0	−	−

*(−) No growth; (+) slight growth; (++) moderate growth; (+++) optimal growth. All the bifidobacterial strains assayed showed the same level of inhibition.

**Table 4:** CLA and CLNA production (*μ*g mL^−1^) and conversion (%) of LA and LNA by *B. breve* strains when growing in skim milk. Conversion calculated as ∑CLA/(LA + ∑CLA) × 100 and ∑CLNA/(LNA + ∑CLNA) × 100, respectively, based on the results of GC.

Strains	CLA	*cis 9 trans 11* (% CLA)	LA conversion (%)	CLNA	*cis 9 trans 11 cis 15 * (% CLNA)	LNA conversion (%)
*B*. *breve* ZL12-28	75.0 ± 9.5 (a)	83.6 ± 5.3 (a)	31.3 ± 14 (a)	243.7 ± 39.8 (a)	93.6 ± 0.8 (a)	96.5 ± 1.2 (a)
*B*. *breve* 29M2	95.0 ± 12.4 (a)	87.0 ± 6.6 (a)	41.6 ± 11 (a)	219.8 ± 30 (a)	90.4 ± 4.2 (a)	94.0 ± 6.5 (a)
*B*. *breve* M7-70	75.9 ± 6.1 (a)	83.4 ± 4.1 (a)	29.6 ± 12 (a)	210.1 ± 23.8 (a)	90.9 ± 1.9 (a)	97.0 ± 1.4 (a)

Values are means of triplicate experiments and standard deviation (±SD).

Means in the same column with different lowercase letters are significant at *P* < 0.05.
